# The Femoral Artery in War Surgery

**Published:** 1919

**Authors:** 


**The Femoral Artery in War Surgery.** By James H. Nicoll, M.B., *The British Medical Journal,* November 23, 1918.

Sections of standard works on operative surgery which deal with amputations and with hemorrhage will have to be in large measure rewritten after the war. The femoral artery is the largest peripheral trunk in the body and presents some problems in operative surgery of great importance.

*The control of hemorrhage during the operation of amputation at the hip-joint.* Mr. Huggins, in his recent work " Amputation Stumps ", warmly advocates the " anterior racket " variety of operation, with ligature of the femoral vessels as the first step. It renders the surgeon independent of skilled assistance, and removes all risk of serious loss of blood. Among the various methods described in textbooks this one seems to present most advantages.

*The control of hemorrhage in amputation of any portion of the lower limb below the upper third of the thigh.* In such operations one of three methods may be used to control hemorrhage: the tourniquet, manual compression of the femoral artery, or temporary ligature of that vessel, though this last method has little field in war surgery.

The tourniquet is in universal use at the fighting fronts, but it presents great dangers and disadvantages, for it greatly interferes with the nutrition of the wounded tissues. Digital compression of the femoral artery against the pubic ramus is much to be preferred. It may be performed by an orderly, a nurse or a student. Three points are of importance : standing on the outer side of the limb, fixing the thumbs for counter-pressure on the postero-external aspect of the ilium, below the level of the anterior superior spine, and pressing the fingers on the vessel just below Poupart's ligament.

*Prevention of secondary hemorrhage in "open" amputation stumps in the thigh.* Guillotine stumps, flap stumps awaiting secondary suture, and stumps in which the sutures have cut out are often suppurating and sloughing. Hemorrhage is a constant risk after the first week, and it should be prevented by ligature of the common femoral vessels in the case of any stump down to the supracondylar level. In leg stumps ligature of the femoral carries a great risk of resulting gangrene and the need for it, in such cases, is less clamant than in thigh stumps. Fortunately hemorrhage is never so rapid that the assistance of the surgeons cannot be got in time.

